# Unexplained Anemia in the Elderly: Potential Role of Arterial Stiffness

**DOI:** 10.3389/fphys.2016.00485

**Published:** 2016-10-25

**Authors:** David Montero, Candela Diaz-Cañestro, Andreas Flammer, Carsten Lundby

**Affiliations:** ^1^Department of Cardiology, University Heart Center, University Hospital ZurichZurich, Switzerland; ^2^Center for Molecular Cardiology, University of ZurichZurich, Switzerland; ^3^Zurich Center for Integrative Human Physiology, Institute of Physiology, University of ZurichZurich, Switzerland

**Keywords:** arterial stiffness, erythropoietin, blood volume, anemia

## Introduction

Anemia, as defined by hemoglobin concentration < 12.0 and < 13.0 g/dl in women and men, respectively, affects more than 150 million elderly people worldwide and even when mild is associated with increased disability, morbidity, and mortality (Ezekowitz et al., 2003; Penninx et al., 2004; De Benoist et al., 2008). The causes of anemia remain unresolved in approximately one-third of anemic older individuals, commonly referred as unexplained anemia in the elderly (UAE; Guralnik et al., 2004). As most types of anemia, UAE is normocytic and its prevalence increases with advancing age (Guralnik et al., 2004). UAE is uniquely characterized by low plasma erythropoietin (EPO) concentration compared to the other forms of anemia (Ferrucci et al., 2007; Artz and Thirman, 2011). Thus, provided that EPO is mainly synthesized in the kidneys and its clearance/degradation is not enhanced, UAE may be primarily determined by factors affecting renal EPO production and/or hemodilution (Lundby et al., 2014; de Seigneux et al., 2016). These factors are yet to be elucidated. Of note, while hemoglobin concentration is typically employed to diagnose anemia, true (non-hemodilutional) anemia is defined as a decrease in the total volume of red blood cells (RBCV) and/or hemoglobin mass. Herein, we present arguments to support the contention that a fundamental hallmark of vascular aging, arterial stiffness (AS), might be a primary etiological determinant of low EPO concentration and RBCV in UAE via the impairment of mechanisms regulating kidney perfusion, EPO production as well as blood volume. This viewpoint postulates that AS is augmented in UAE patients compared with healthy non-anemic elderly individuals. While direct proof is not available at present, independent lines of evidence demonstrate that UAE is characterized by alterations in biochemical, hematological, and physiological parameters consistently associated with AS (Ferrucci et al., 2007; Robinson et al., 2007; Artz et al., 2014; Malahfji et al., 2014), whereof some are described in detail below.

## Association between arterial stiffness (AS) and the total volume of red blood cells (RBCV)

AS characterizes the reduced capacity of an artery to expand and recoil in response to changes in blood pressure. When assessed in central elastic arteries (e.g., carotid), AS independently predicts incident cardiovascular events, kidney disease, and all-cause mortality (Hashimoto and Ito, 2011; van Sloten et al., 2014). Sedentary aging is inexorably associated with a linear or exponential increase in AS (Avolio et al., 1983; Mitchell et al., 2004; McEniery et al., 2005), possibly reflecting the gradual fragmentation of elastin fibers due to repetitive cyclic stress as well as the simultaneous accumulation of collagen fibers in the media layer of elastic arteries (O'Rourke and Hashimoto, 2007; Camici et al., 2015). Nonetheless, there is substantial variability in the degree of arterial stiffening with advancing age (Avolio et al., 1983). For instance, older individuals regularly involved in endurance training may present similar low levels of AS than healthy young controls (Vaitkevicius et al., 1993; Tanaka et al., 1998). These elderly endurance-trained individuals also exhibit a preserved blood volume and RBCV, both declining with age in sedentary adults (Jones et al., 1997). The increase in RBCV commonly observed with endurance training in healthy individuals has been proposed to be partially related to frequent reductions in central (intrathoracic) venous pressure (CVP) stimulating EPO synthesis after each exercise bout (Kirsch et al., 2005; Montero et al., 2015), in line with the basal (non-hypoxic) regulation of EPO production as outlined in the next section. Parenthetically, RBCV is the main determinant of cardiorespiratory capacity (Montero et al., 2015), which in turn is inversely associated with AS irrespective of age status (Vaitkevicius et al., 1993; Ferreira et al., 2003; Kitzman et al., 2013). Thus, it is reasonable to expect a negative relationship between AS and RBCV, and this has been recently confirmed experimentally (Montero et al., 2016a). Specifically, carotid AS and carotid-ankle pulse wave velocity are strongly and negatively associated (*r* ≥ −0.62) with total hemoglobin mass, RBCV, and blood volume independently of body weight and gender in healthy adults (Montero et al., 2016a). In addition, augmentation index, an indirect measure of AS, has been negatively related to blood hemoglobin concentration in a cohort of individuals presenting with diabetes alone or along with anemia (Demir et al., 2015). It should be noted, however, that statistical associations do not necessarily infer strict causal relationships. Both AS and impaired erythropoiesis could be caused by a common underlying process, e.g., an age-related increase in circulating pro-inflammatory cytokines (Makipour et al., 2008; Artz et al., 2014), which may hinder proliferation and differentiation of erythroid progenitor cells independently of circulating EPO levels (Means, 1995; Selleri et al., 1996). Acknowledging this uncertainty, multiple linear correlative evidence impelled us to ponder whether AS-related mechanisms may adversely influence the regulation of erythropoiesis and explain, at least in part, the uncertain pathophysiology of UAE.

## Regulation of erythropoietin (EPO) synthesis through blood volume feedback mechanisms

It is beyond dispute that tissue hypoxia in peritubular fibroblast-like cells of the renal cortex stimulates the synthesis of EPO upon stabilization of hypoxia-inducible factor-2α (Jelkmann, 2011). However, additional non-hypoxic feedback mechanisms must also regulate EPO production since arterial O_2_ partial pressure and renal blood flow/O_2_ consumption seldom fluctuate to values required to substantially increase the synthesis of EPO in healthy individuals (Halperin et al., 2006; Perez-Padilla et al., 2006). Among potential non-hypoxic factors related to erythropoiesis, EPO concentrations run inversely to CVP, a variable reflecting the filling state of the cardiovascular system (Ehmke et al., 1995; Gunga et al., 1996; Breymann et al., 2000). In this respect, early animal studies observed a 1.5-fold acute increase in plasma EPO levels following a reduction of blood volume (20%) and CVP while hematocrit was unaltered (Ehmke et al., 1995). In humans, hypervolemic hemodilution increasing CVP paralleled a decrease in circulating EPO seemingly beyond the dilution effect (Breymann et al., 2000). Recently, plasma EPO concentration was found increased independent of hemoconcentration, with moderate head-up tilt redistributing blood toward the lower limbs and thereby reducing CVP (Montero et al., 2016b). Overall, these studies suggest that CVP *per-se* could be a regulator of EPO production.

The specific mechanism(s) explaining the link between CVP and EPO synthesis has yet to be established. Nonetheless, changes in EPO concentration in response to variations in CVP resembles the pattern of hormones governing blood volume such as those pertaining to the renin-angiotensin-aldosterone system (RAAS) as well as natriuretic peptides and vasopressin (VPN; Gauer and Henry, 1963). These hormones regulate blood volume through feedback loops including veno-atrial and arterial baroreceptors (Gauer and Henry, 1963). Interestingly, angiotensin II (ANGII) and VPN stimulate EPO synthesis *in vivo* independently of kidney tissue hypoxia (Engel and Pagel, 1995; Kim et al., 2014), and are augmented when CVP is decreased (Segar and Moore, 1968; Egan et al., 1984; Bie et al., 1986; Sander-Jensen et al., 1986). Accordingly, the production of EPO has been proposed to be regulated under basal (non-kidney hypoxic) conditions by blood volume-regulating hormones (Kirsch et al., 2005). Hence, EPO synthesis and therefore erythropoiesis could be affected by alterations in hemodynamic and endocrine feedback pathways that control blood volume.

## Potential mechanisms linking arterial stiffness (AS) and low erythropoietin (EPO) concentration in unexplained anemia in the elderly (UAE) (Figure 1)

### Altered baroreflex sensitivity (BRS) and stimulated release of blood volume-regulating hormones

Baroreflex sensitivity (BRS) declines progressively with age and is primarily associated with the compliance of central elastic arteries in which high-pressure baroreceptors are located (aortic arch and carotid arteries; Monahan et al., 2001a). This is only natural given that baroreceptor firing rate is proportional to changes in arterial circumference (Kingwell et al., 1995). Monahan et al. demonstrated that carotid compliance independently explains the majority of the variance of BRS across the lifespan (Monahan et al., 2001a). Moreover, age-related reduction in BRS is largely attenuated by endurance training (Monahan et al., 2000, 2001a,b), which concurs with the aforementioned observations regarding AS and hematological variables. Taking into account that arterial baroreceptors control the secretion of blood volume-regulating hormones such as VPN (Thrasher, 1994; Thrasher and Keil, 1998), this could be affected by AS. In this regard, increased arterial pulse pressure, a common correlate of AS and impaired BRS (Virtanen et al., 2004), decreases the release of VPN even in the absence of changes in mean arterial pressure (Gabrielsen et al., 2000). In this line, aging has been associated with a blunted VPN release in response to conditions associated with reduced CVP such as acute post-exercise hypotension (Kirsch et al., 1986; Halliwill et al., 2000; Keck et al., 2000). Importantly, the increase in EPO concentration induced by decreasing CVP is abolished when adjusted for the concomitant increase in copeptin (Montero et al., 2016b), a marker of VPN (Morgenthaler et al., 2008). Indeed, changes in copeptin with experimental manipulation of CVP independently explained simultaneously occurring changes in EPO concentration in healthy adults (Montero et al., 2016b). Furthermore, VPN administration is associated with increased plasma EPO levels and red cell mass in hypopituitaric patients (Jepson et al., 1968). As for the underlying mechanisms, animal studies demonstrated that VPN directly induces EPO secretion through the activation of V1a receptors (Engel and Pagel, 1995), which are expressed in the renal cortex and medulla and mediate the vasopressor effect of VPN (Gózdz et al., 2002; Koshimizu et al., 2006). Therefore, whilst speculative, the characteristic low EPO concentration in UAE could be attributed, at least in part, to reduced VPN release secondary to AS and impaired BRS. The rate of VPN delivery to kidney target cells depends, in addition to plasma VPN concentration and plasma volume, on renal blood flow, which leads us to the next potential mechanisms linking AS and UAE.

**Figure 1:**
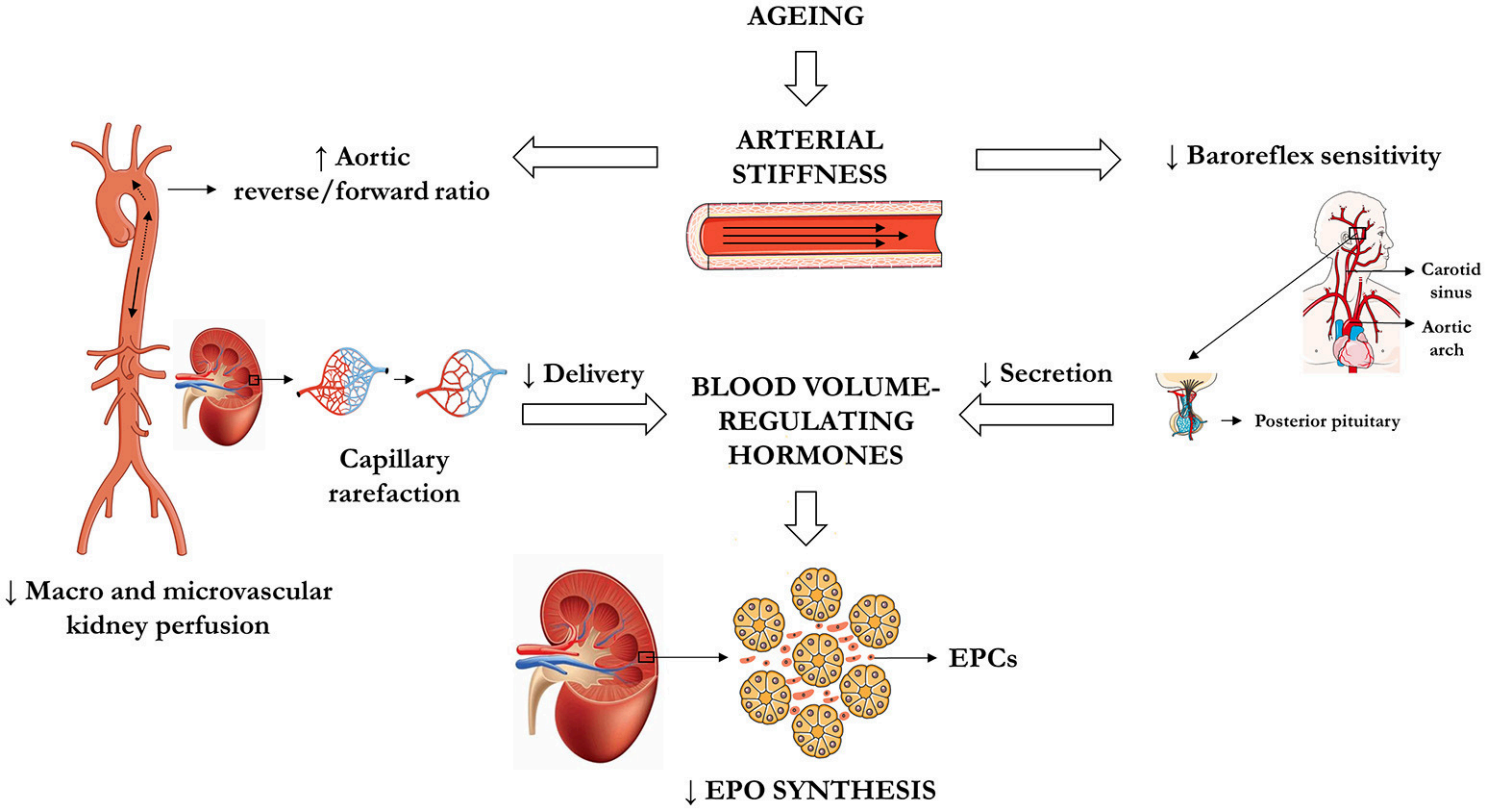
**Central hypothesis linking arterial stiffness in central elastic arteries and low EPO concentration in UAE**. EPCs, epo-producing cells; EPO, erythropoietin; UAE, unexplained anemia in the elderly.

### Altered renal blood flow

The relationship between AS and kidney function is amply acknowledged in the literature (Safar et al., 2004; O'Rourke and Safar, 2005; Ford et al., 2010). Central to this relationship is the inherent loss of the elastic recoil (Windkessel function) in the descending aorta, which supplies blood to the kidneys. The higher the degree of aortic stiffening, the greater is the aortic upstream (reverse) flow in early diastole (Hashimoto and Ito, 2013). Kidney perfusion and thereby glomerular filtration rate (GFR) are reduced according to the increase in aortic flow reversal, independent of cardiac output (Hashimoto and Ito, 2015). In fact, the aortic reverse/forward flow ratio is the strongest determinant of estimated GFR as well as renal blood flow, and mediates the association of AS with GFR in hypertensive patients (Hashimoto and Ito, 2015). In individuals with UAE, a low estimated GFR (≤ 60 mL·min·1.73 m^2^) seems prevalent (Robinson et al., 2007; Artz et al., 2014), possibly reflecting a concomitant reduction in renal blood flow considering its expected association with GFR (Lebkowska et al., 2007; Hashimoto and Ito, 2015). Consequently, AS may lead to a specific reduction of blood flow to the kidneys, thus diminish the delivery of blood-volume regulating hormones and as a direct consequence hereof affect EPO synthesis.

### Impaired tissue perfusion

One of the most clinically relevant outcomes derived from AS is the increase in blood pressure pulse amplitude (Struijker-Boudier, 2009). The augmented pulsatility primarily impacts on the smallest arteries and arterioles, i.e., the resistance vasculature. When the increase in pulse pressure is maintained, adverse phenotypic changes are commonly observed, particularly in vascular beds characterized by low resistance such as the renal microcirculation (Mitchell, 2008; Woodard et al., 2015). These microvascular alterations include increased media-to-lumen ratio, decreased arteriolar diameter, and capillary rarefaction, among others (Levy et al., 2001). In this respect, an increased vascular resistance and reduced arterial volume in the renal cortex explain the negative association between renal artery pulsatility index (PI) and GFR in older adults (Woodard et al., 2015). Furthermore, the association between AS, pulse pressure and microvascular abnormalities is not a one-way relationship, in that the latter may contribute to AS and pulse pressure, leading to a vicious circle (Struijker-Boudier, 2009). Not surprisingly, impaired tissue perfusion is common to diseases characterized by AS such as hypertension, obesity, diabetes mellitus, and aging *per-se* (Delp et al., 1998; Levy et al., 2008). Hence, AS could cause a specific reduction in the microvascular perfusion of peritubular fibroblast-like cells of the renal cortex (i.e., kidney EPO-producing cells; Jelkmann, 2011), thus being another contributing factor to low EPO concentration in UAE.

## Conclusion

Among all types of anemia, the most prevalent, UAE, remain currently unexplained (Artz and Thirman, 2011). In this viewpoint, we have highlighted the plausibly overlooked role of AS, a well-known feature of the vascular aging process, as a primary contributing factor to the typical low EPO concentration observed in UAE. Underlying mechanisms are proposed to include the impairment of endocrine feedback pathways governing the basal regulation of kidney EPO synthesis as well as renal perfusion. In particular, AS in central elastic arteries leading to baroreceptor dysfunction may hinder the release of blood volume-regulating hormones directly stimulating EPO synthesis. Moreover, AS is closely linked with the reduction of renal blood flow and microvascular perfusion contributing to impaired hormone delivery to EPO-producing cells. While the rationale underlying the potential impact of AS on the etiology of UAE is based on fundamental hemodynamic and endocrine (dys) regulation, its specific relevance to UAE will have to be established in future studies. These may yield relevant insights for novel and effective therapeutic targets in the treatment of UAE.

## Author contributions

DM drafted the manuscript. DM, CD, AF, and CL critically revised the manuscript for important intellectual content, and approved the final the version to be published.

### Conflict of interest statement

The authors declare that the research was conducted in the absence of any commercial or financial relationships that could be construed as a potential conflict of interest.
